# Supplementary material for: Modeling glioblastoma heterogeneity as a dynamic network of cell states
Source: Mol Syst Biol. 2021 Sep 16;17(9):e10105. doi: 10.15252/msb.202010105 (PMC8444284; doi:10.15252/msb.202010105)
Supplement: Supplementary file 5 — Source Data for Figure 3 [file MSB-17-e10105-s001.zip › Figure3A_sourcedata/GSEA_3065/hallmarks_state2.GseaPreranked.1623416269049/HALLMARK_MYC_TARGETS_V1.html]

Details for gene set HALLMARK\_MYC\_TARGETS\_V1[GSEA]

|  || Dataset | state2 |
| Phenotype | NoPhenotypeAvailable |
| Upregulated in class | na\_pos |
| GeneSet | HALLMARK\_MYC\_TARGETS\_V1 |
| Enrichment Score (ES) | 0.7800272 |
| Normalized Enrichment Score (NES) | 2.8026154 |
| Nominal p-value | 0.0 |
| FDR q-value | 0.0 |
| FWER p-Value | 0.0 |
Table: GSEA Results Summary

  

Fig 1: Enrichment plot: HALLMARK\_MYC\_TARGETS\_V1      
 Profile of the Running ES Score & Positions of GeneSet Members on the Rank Ordered List

  

| PROBE | GENE SYMBOL | GENE\_TITLE | RANK IN GENE LIST | RANK METRIC SCORE | RUNNING ES | CORE ENRICHMENT || 1 | TYMS |  |  | 5 | 0.792 | 0.0227 | Yes |
| 2 | MCM7 |  |  | 13 | 0.626 | 0.0404 | Yes |
| 3 | PCNA |  |  | 16 | 0.595 | 0.0576 | Yes |
| 4 | MAD2L1 |  |  | 20 | 0.563 | 0.0739 | Yes |
| 5 | DUT |  |  | 24 | 0.552 | 0.0898 | Yes |
| 6 | DEK |  |  | 31 | 0.526 | 0.1046 | Yes |
| 7 | KPNA2 |  |  | 50 | 0.472 | 0.1165 | Yes |
| 8 | RANBP1 |  |  | 65 | 0.430 | 0.1276 | Yes |
| 9 | RRM1 |  |  | 72 | 0.412 | 0.1391 | Yes |
| 10 | SNRPD1 |  |  | 74 | 0.408 | 0.1510 | Yes |
| 11 | MCM4 |  |  | 79 | 0.399 | 0.1622 | Yes |
| 12 | MCM5 |  |  | 83 | 0.394 | 0.1735 | Yes |
| 13 | HNRNPD |  |  | 88 | 0.390 | 0.1845 | Yes |
| 14 | CDC45 |  |  | 95 | 0.377 | 0.1949 | Yes |
| 15 | RAN |  |  | 98 | 0.375 | 0.2057 | Yes |
| 16 | RFC4 |  |  | 126 | 0.339 | 0.2128 | Yes |
| 17 | PTGES3 |  |  | 128 | 0.336 | 0.2226 | Yes |
| 18 | CDK4 |  |  | 131 | 0.333 | 0.2321 | Yes |
| 19 | CCNA2 |  |  | 135 | 0.331 | 0.2415 | Yes |
| 20 | PA2G4 |  |  | 137 | 0.329 | 0.2511 | Yes |
| 21 | USP1 |  |  | 138 | 0.329 | 0.2608 | Yes |
| 22 | SET |  |  | 139 | 0.328 | 0.2704 | Yes |
| 23 | SRSF3 |  |  | 140 | 0.327 | 0.2800 | Yes |
| 24 | SNRPG |  |  | 144 | 0.322 | 0.2891 | Yes |
| 25 | MCM6 |  |  | 149 | 0.311 | 0.2979 | Yes |
| 26 | CBX3 |  |  | 152 | 0.308 | 0.3067 | Yes |
| 27 | SRSF7 |  |  | 155 | 0.306 | 0.3155 | Yes |
| 28 | HSPD1 |  |  | 176 | 0.294 | 0.3219 | Yes |
| 29 | PPIA |  |  | 189 | 0.287 | 0.3291 | Yes |
| 30 | CDC20 |  |  | 193 | 0.284 | 0.3371 | Yes |
| 31 | HSPE1 |  |  | 211 | 0.276 | 0.3434 | Yes |
| 32 | SRSF2 |  |  | 213 | 0.275 | 0.3514 | Yes |
| 33 | NME1 |  |  | 214 | 0.274 | 0.3594 | Yes |
| 34 | LSM2 |  |  | 219 | 0.272 | 0.3670 | Yes |
| 35 | UBA2 |  |  | 223 | 0.268 | 0.3745 | Yes |
| 36 | SNRPD3 |  |  | 226 | 0.265 | 0.3821 | Yes |
| 37 | MCM2 |  |  | 228 | 0.262 | 0.3897 | Yes |
| 38 | SERBP1 |  |  | 230 | 0.262 | 0.3973 | Yes |
| 39 | ILF2 |  |  | 240 | 0.258 | 0.4039 | Yes |
| 40 | LSM7 |  |  | 242 | 0.255 | 0.4113 | Yes |
| 41 | VBP1 |  |  | 245 | 0.254 | 0.4185 | Yes |
| 42 | HNRNPR |  |  | 269 | 0.244 | 0.4232 | Yes |
| 43 | NHP2 |  |  | 274 | 0.242 | 0.4299 | Yes |
| 44 | CCT5 |  |  | 279 | 0.242 | 0.4366 | Yes |
| 45 | COX5A |  |  | 308 | 0.229 | 0.4402 | Yes |
| 46 | BUB3 |  |  | 311 | 0.228 | 0.4467 | Yes |
| 47 | ERH |  |  | 312 | 0.228 | 0.4534 | Yes |
| 48 | HNRNPA3 |  |  | 320 | 0.226 | 0.4593 | Yes |
| 49 | PPM1G |  |  | 324 | 0.225 | 0.4656 | Yes |
| 50 | POLD2 |  |  | 330 | 0.223 | 0.4716 | Yes |
| 51 | POLE3 |  |  | 332 | 0.223 | 0.4780 | Yes |
| 52 | TRIM28 |  |  | 334 | 0.222 | 0.4844 | Yes |
| 53 | KPNB1 |  |  | 350 | 0.216 | 0.4892 | Yes |
| 54 | NPM1 |  |  | 351 | 0.216 | 0.4955 | Yes |
| 55 | EIF1AX |  |  | 356 | 0.215 | 0.5014 | Yes |
| 56 | SNRPA1 |  |  | 361 | 0.214 | 0.5073 | Yes |
| 57 | GLO1 |  |  | 363 | 0.214 | 0.5134 | Yes |
| 58 | RPS2 |  |  | 368 | 0.212 | 0.5192 | Yes |
| 59 | C1QBP |  |  | 378 | 0.209 | 0.5244 | Yes |
| 60 | CDK2 |  |  | 382 | 0.208 | 0.5302 | Yes |
| 61 | NAP1L1 |  |  | 387 | 0.207 | 0.5358 | Yes |
| 62 | NOP56 |  |  | 396 | 0.201 | 0.5409 | Yes |
| 63 | CCT4 |  |  | 405 | 0.199 | 0.5459 | Yes |
| 64 | RNPS1 |  |  | 410 | 0.198 | 0.5513 | Yes |
| 65 | PSMA7 |  |  | 431 | 0.193 | 0.5548 | Yes |
| 66 | ACP1 |  |  | 433 | 0.193 | 0.5603 | Yes |
| 67 | RPLP0 |  |  | 451 | 0.190 | 0.5641 | Yes |
| 68 | TXNL4A |  |  | 467 | 0.186 | 0.5679 | Yes |
| 69 | SSB |  |  | 478 | 0.184 | 0.5722 | Yes |
| 70 | HDAC2 |  |  | 479 | 0.184 | 0.5776 | Yes |
| 71 | RSL1D1 |  |  | 484 | 0.183 | 0.5826 | Yes |
| 72 | TCP1 |  |  | 490 | 0.182 | 0.5874 | Yes |
| 73 | YWHAQ |  |  | 498 | 0.182 | 0.5920 | Yes |
| 74 | PSMB3 |  |  | 502 | 0.180 | 0.5969 | Yes |
| 75 | SYNCRIP |  |  | 503 | 0.180 | 0.6022 | Yes |
| 76 | LDHA |  |  | 506 | 0.179 | 0.6073 | Yes |
| 77 | HNRNPA2B1 |  |  | 510 | 0.178 | 0.6122 | Yes |
| 78 | YWHAE |  |  | 511 | 0.178 | 0.6174 | Yes |
| 79 | TRA2B |  |  | 515 | 0.178 | 0.6223 | Yes |
| 80 | CYC1 |  |  | 520 | 0.178 | 0.6271 | Yes |
| 81 | VDAC3 |  |  | 528 | 0.176 | 0.6316 | Yes |
| 82 | SNRPD2 |  |  | 551 | 0.172 | 0.6342 | Yes |
| 83 | CCT2 |  |  | 556 | 0.171 | 0.6388 | Yes |
| 84 | HDGF |  |  | 570 | 0.168 | 0.6424 | Yes |
| 85 | HPRT1 |  |  | 573 | 0.168 | 0.6471 | Yes |
| 86 | IMPDH2 |  |  | 575 | 0.167 | 0.6519 | Yes |
| 87 | PCBP1 |  |  | 582 | 0.166 | 0.6561 | Yes |
| 88 | RPS3 |  |  | 598 | 0.164 | 0.6593 | Yes |
| 89 | CNBP |  |  | 599 | 0.164 | 0.6641 | Yes |
| 90 | SSBP1 |  |  | 606 | 0.163 | 0.6682 | Yes |
| 91 | XRCC6 |  |  | 641 | 0.158 | 0.6692 | Yes |
| 92 | PSMD7 |  |  | 658 | 0.155 | 0.6720 | Yes |
| 93 | UBE2L3 |  |  | 671 | 0.153 | 0.6752 | Yes |
| 94 | RPS5 |  |  | 679 | 0.152 | 0.6789 | Yes |
| 95 | PSMD8 |  |  | 690 | 0.151 | 0.6823 | Yes |
| 96 | VDAC1 |  |  | 713 | 0.149 | 0.6843 | Yes |
| 97 | PGK1 |  |  | 726 | 0.146 | 0.6873 | Yes |
| 98 | FBL |  |  | 738 | 0.146 | 0.6904 | Yes |
| 99 | ABCE1 |  |  | 746 | 0.144 | 0.6939 | Yes |
| 100 | SNRPA |  |  | 754 | 0.143 | 0.6973 | Yes |
| 101 | EIF4A1 |  |  | 765 | 0.142 | 0.7004 | Yes |
| 102 | EEF1B2 |  |  | 773 | 0.142 | 0.7038 | Yes |
| 103 | PSMA4 |  |  | 797 | 0.138 | 0.7054 | Yes |
| 104 | PHB |  |  | 802 | 0.138 | 0.7090 | Yes |
| 105 | RPS6 |  |  | 819 | 0.136 | 0.7113 | Yes |
| 106 | PHB2 |  |  | 838 | 0.134 | 0.7133 | Yes |
| 107 | HNRNPC |  |  | 850 | 0.133 | 0.7160 | Yes |
| 108 | TFDP1 |  |  | 874 | 0.130 | 0.7173 | Yes |
| 109 | HNRNPA1 |  |  | 876 | 0.130 | 0.7210 | Yes |
| 110 | PRDX3 |  |  | 877 | 0.130 | 0.7249 | Yes |
| 111 | PRPF31 |  |  | 901 | 0.128 | 0.7261 | Yes |
| 112 | EIF4H |  |  | 924 | 0.126 | 0.7274 | Yes |
| 113 | RPL6 |  |  | 968 | 0.122 | 0.7264 | Yes |
| 114 | NDUFAB1 |  |  | 970 | 0.122 | 0.7299 | Yes |
| 115 | RAD23B |  |  | 973 | 0.122 | 0.7332 | Yes |
| 116 | CCT7 |  |  | 1029 | 0.117 | 0.7307 | Yes |
| 117 | EIF4E |  |  | 1038 | 0.116 | 0.7333 | Yes |
| 118 | EIF2S1 |  |  | 1051 | 0.115 | 0.7354 | Yes |
| 119 | RPL14 |  |  | 1057 | 0.115 | 0.7382 | Yes |
| 120 | PSMB2 |  |  | 1061 | 0.114 | 0.7412 | Yes |
| 121 | HDDC2 |  |  | 1071 | 0.113 | 0.7436 | Yes |
| 122 | GNL3 |  |  | 1084 | 0.112 | 0.7456 | Yes |
| 123 | EXOSC7 |  |  | 1100 | 0.112 | 0.7472 | Yes |
| 124 | NOLC1 |  |  | 1104 | 0.111 | 0.7502 | Yes |
| 125 | PSMA2 |  |  | 1125 | 0.110 | 0.7512 | Yes |
| 126 | EIF3B |  |  | 1141 | 0.109 | 0.7528 | Yes |
| 127 | PSMA1 |  |  | 1149 | 0.108 | 0.7552 | Yes |
| 128 | MRPL9 |  |  | 1152 | 0.108 | 0.7582 | Yes |
| 129 | SRM |  |  | 1160 | 0.107 | 0.7606 | Yes |
| 130 | MRPL23 |  |  | 1176 | 0.106 | 0.7621 | Yes |
| 131 | PRPS2 |  |  | 1221 | 0.103 | 0.7603 | Yes |
| 132 | CTPS1 |  |  | 1223 | 0.103 | 0.7633 | Yes |
| 133 | SNRPB2 |  |  | 1232 | 0.102 | 0.7654 | Yes |
| 134 | PSMC4 |  |  | 1237 | 0.102 | 0.7680 | Yes |
| 135 | SRPK1 |  |  | 1240 | 0.102 | 0.7707 | Yes |
| 136 | EIF2S2 |  |  | 1245 | 0.102 | 0.7733 | Yes |
| 137 | STARD7 |  |  | 1272 | 0.100 | 0.7734 | Yes |
| 138 | COPS5 |  |  | 1302 | 0.098 | 0.7732 | Yes |
| 139 | SRSF1 |  |  | 1315 | 0.097 | 0.7747 | Yes |
| 140 | RPL22 |  |  | 1321 | 0.097 | 0.7770 | Yes |
| 141 | HNRNPU |  |  | 1369 | 0.094 | 0.7747 | Yes |
| 142 | GSPT1 |  |  | 1377 | 0.093 | 0.7767 | Yes |
| 143 | CCT3 |  |  | 1432 | 0.090 | 0.7735 | Yes |
| 144 | CLNS1A |  |  | 1445 | 0.090 | 0.7748 | Yes |
| 145 | PSMD1 |  |  | 1468 | 0.088 | 0.7751 | Yes |
| 146 | DDX21 |  |  | 1486 | 0.087 | 0.7758 | Yes |
| 147 | SMARCC1 |  |  | 1516 | 0.086 | 0.7752 | Yes |
| 148 | RPL18 |  |  | 1526 | 0.085 | 0.7767 | Yes |
| 149 | DHX15 |  |  | 1565 | 0.083 | 0.7750 | Yes |
| 150 | G3BP1 |  |  | 1566 | 0.083 | 0.7775 | Yes |
| 151 | RRP9 |  |  | 1583 | 0.082 | 0.7782 | Yes |
| 152 | PABPC1 |  |  | 1589 | 0.082 | 0.7800 | Yes |
| 153 | PSMD14 |  |  | 1627 | 0.080 | 0.7784 | No |
| 154 | NCBP1 |  |  | 1652 | 0.079 | 0.7781 | No |
| 155 | ETF1 |  |  | 1749 | 0.075 | 0.7699 | No |
| 156 | SLC25A3 |  |  | 1824 | 0.071 | 0.7640 | No |
| 157 | XPO1 |  |  | 1871 | 0.070 | 0.7611 | No |
| 158 | PSMC6 |  |  | 1874 | 0.070 | 0.7629 | No |
| 159 | EIF4G2 |  |  | 1887 | 0.069 | 0.7636 | No |
| 160 | ODC1 |  |  | 1913 | 0.068 | 0.7629 | No |
| 161 | RPS10 |  |  | 1973 | 0.066 | 0.7585 | No |
| 162 | PABPC4 |  |  | 2029 | 0.063 | 0.7544 | No |
| 163 | FAM120A |  |  | 2030 | 0.063 | 0.7563 | No |
| 164 | EIF3J |  |  | 2081 | 0.062 | 0.7527 | No |
| 165 | AIMP2 |  |  | 2087 | 0.061 | 0.7539 | No |
| 166 | SF3B3 |  |  | 2121 | 0.060 | 0.7521 | No |
| 167 | RUVBL2 |  |  | 2141 | 0.060 | 0.7518 | No |
| 168 | AP3S1 |  |  | 2167 | 0.059 | 0.7509 | No |
| 169 | RPL34 |  |  | 2224 | 0.056 | 0.7465 | No |
| 170 | RACK1 |  |  | 2226 | 0.056 | 0.7480 | No |
| 171 | APEX1 |  |  | 2239 | 0.056 | 0.7483 | No |
| 172 | PSMD3 |  |  | 2243 | 0.056 | 0.7496 | No |
| 173 | CUL1 |  |  | 2498 | 0.048 | 0.7236 | No |
| 174 | TOMM70 |  |  | 2558 | 0.046 | 0.7185 | No |
| 175 | CSTF2 |  |  | 2752 | 0.040 | 0.6989 | No |
| 176 | NOP16 |  |  | 2768 | 0.040 | 0.6984 | No |
| 177 | IFRD1 |  |  | 2840 | 0.038 | 0.6918 | No |
| 178 | EIF3D |  |  | 2985 | 0.034 | 0.6773 | No |
| 179 | XPOT |  |  | 3155 | 0.031 | 0.6599 | No |
| 180 | SF3A1 |  |  | 3178 | 0.030 | 0.6584 | No |
| 181 | TUFM |  |  | 3799 | 0.019 | 0.5919 | No |
| 182 | MYC |  |  | 3842 | 0.018 | 0.5879 | No |
| 183 | PWP1 |  |  | 4321 | 0.010 | 0.5365 | No |
| 184 | HSP90AB1 |  |  | 4656 | 0.005 | 0.5005 | No |
| 185 | MRPS18B |  |  | 4729 | 0.004 | 0.4928 | No |
| 186 | CAD |  |  | 4804 | 0.003 | 0.4849 | No |
| 187 | ORC2 |  |  | 4999 | -0.000 | 0.4639 | No |
| 188 | NCBP2 |  |  | 5108 | -0.002 | 0.4523 | No |
| 189 | TARDBP |  |  | 5402 | -0.007 | 0.4208 | No |
| 190 | GOT2 |  |  | 5473 | -0.008 | 0.4135 | No |
| 191 | UBE2E1 |  |  | 5726 | -0.012 | 0.3866 | No |
| 192 | DDX18 |  |  | 5826 | -0.014 | 0.3763 | No |
| 193 | PRDX4 |  |  | 9151 | -0.178 | 0.0220 | No |
| 194 | CANX |  |  | 9374 | -0.310 | 0.0071 | No |
Table: GSEA details [plain text format]

  

Fig 2: HALLMARK\_MYC\_TARGETS\_V1: Random ES distribution      
 Gene set null distribution of ES for **HALLMARK\_MYC\_TARGETS\_V1**
